# Structural Consequences of Variation in SARS-CoV-2 B.1.1.7

**DOI:** 10.33696/immunology.3.085

**Published:** 2021

**Authors:** David A. Ostrov

**Affiliations:** Department of Pathology, Immunology and Laboratory Medicine, University of Florida College of Medicine, Gainesville, FL, USA

**Keywords:** SARS-CoV-2, Angiotensin Converting Enzyme-2, Mutation, Drug discovery

## Abstract

New globally circulating SARS-CoV-2 strains are causing concern about evolution of virus transmissibility, fitness and immune evasion mechanisms. A variant emerging from the United Kingdom called SARS-CoV-2 VUI 202012/01, or B.1.1.7, is thought to exhibit increased transmissibility that results from replication 4–10 times faster than the original Wuhan virus (Wuhan-Hu-1). Although this property is suspected to result from a specific mutation in the spike glycoprotein, D614G, there are 9 mutations that distinguish the UK variant B.1.1.7 from Wuhan-Hu-1 yet to be evaluated for functional effects. We asked if mutated positions fixed in UK variant B.1.1.7 may be involved in the virus life cycle, or evasion of the immune response, by modeling the UK variant spike protein and conducting structural analysis of mutations on the spike glycoprotein trimer (protomer) complexed to ACE2. Importantly, 4 out of 9 differences between the UK variant B.1.1.7 and Wuhan-Hu-1 spike protein alter direct intermolecular interactions. N501Y increased affinity between the spike protein and ACE2. The mutations at A570D, D614G and S982A reduced contact between individual chains of the trimeric spike protomer, potentially enhancing cleavage into S1 and S2 subunits, dynamic structural rearrangement and host cell fusion mechanisms. These data suggest that combined characteristics of mutations unique to UK variant B.1.1.7 enable high affinity binding to ACE2 and enhanced replication properties. The D614G mutation, associated with enhanced virus transmissibility, opens a potentially druggable structural pocket at the interface between spike glycoprotein subunits S1 and S2.

## Results and Discussion

**Figure 1:**
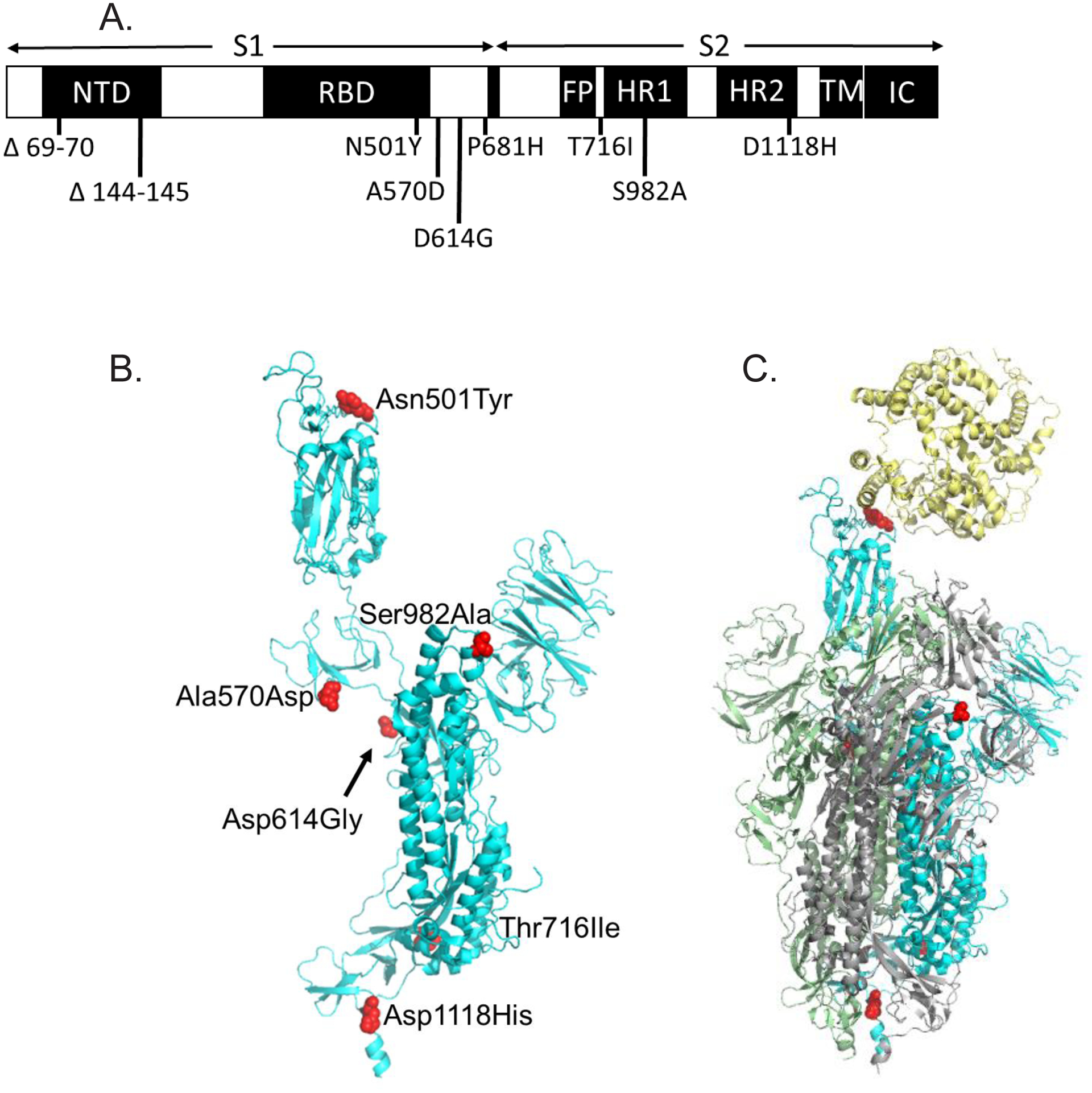
The location of mutated positions that distinguish the United Kingdom variant SARS-CoV-2 B.1.1.7 from Wuhan-Hu-1. **A**. Sites that differ between B.1.1.7 and Wuhan-Hu-1 indicated on the primary structure of the coronavirus spike protein. NTD: N-terminal Domain; RBD: Receptor Binding Domain; FP: Fusion Peptide; HR1: Heptad Repeat 1; HR2: Heptad Repeat 2; TM: Transmembrane anchor; IC: Intracellular tail. **B**. Ribbon diagram model of B.1.1.7 spike glycoprotein based on the structure of the trimeric protomer (PDB 6VSB). Sites that differ between B.1.1.7 and Wuhan-Hu-1 are shown as red spheres. One chain of the spike trimer is shown in blue with one RBD in the up conformation. **C**. Model of the B.1.1.7 spike protein trimer complexed to ACE2. ACE2 is shown in yellow. The spike protein trimer is shown in blue, green and grey. The modeled interaction between ACE2 and the RBD was based on the 2019-nCoV RBD/ACE2 complex (PDB 6M17).

**Figure 2:**
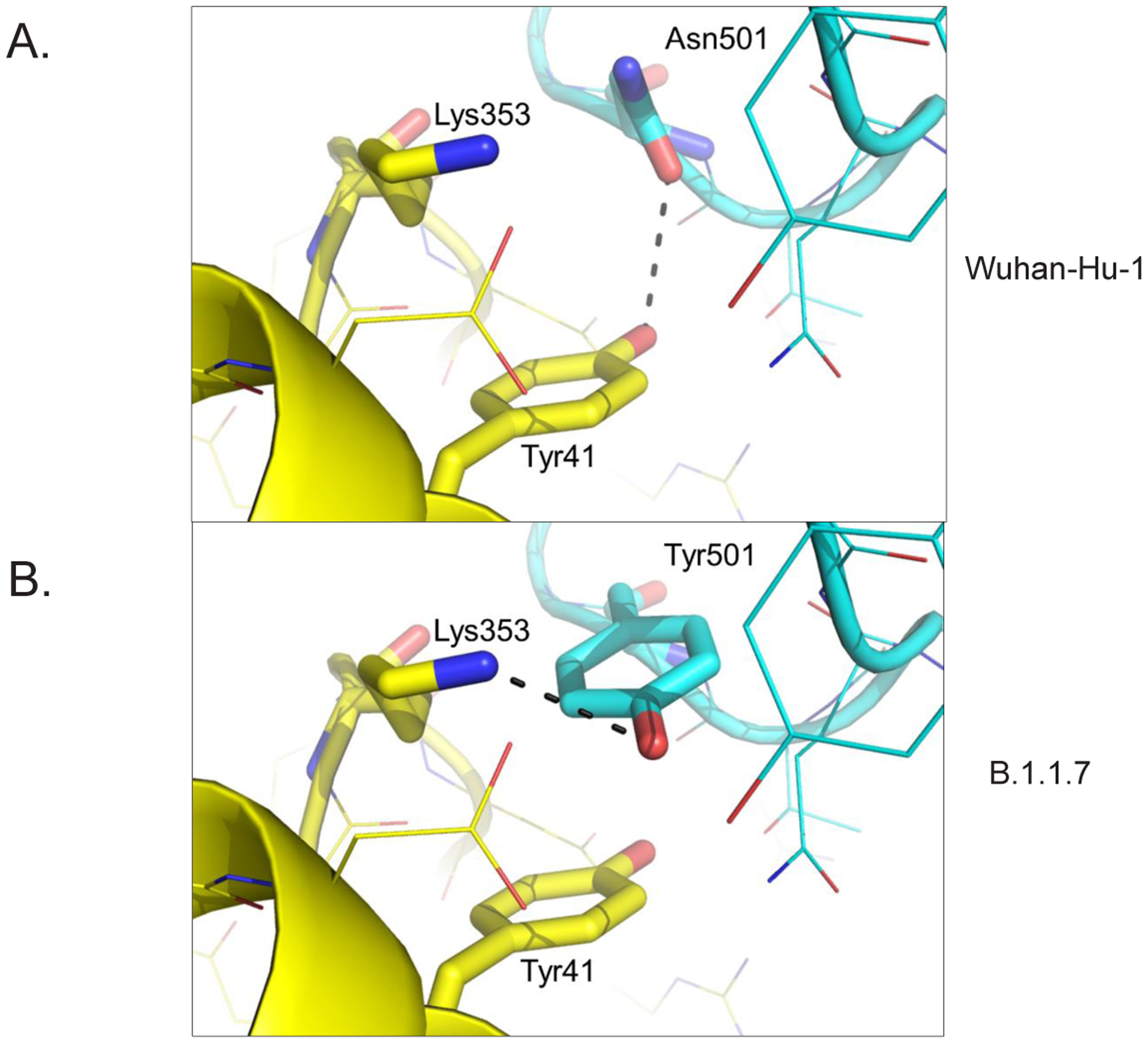
A mutation at position 501 in SARS-CoV-2 B.1.1.7 increases affinity for ACE2 by altering intermolecular interactions. **A**. Asn501 in the spike glycoprotein from Wuhan-Hu-1 (blue) forms a H bond (black dashes) with Tyr41 in ACE2 (yellow). **B**. Tyr501 in SARS-CoV-2 VUI 202012/01 form an aromatic stack with Tyr41 and a H bond with Lys353.

There are 9 sites that differ between SARS-CoV-2 spike glycoproteins from the original Wuhan strain (Wuhan-Hu-1) and UK variant B.1.1.7 [1] (Figure 1, Table 1). There are 2 sites with deletions (positions 69–70, 144–145) and 7 sites with amino acid substitutions (4 positions in the S1 subunit and 3 positions in S2). Most substitutions in the spike protein that distinguish UK variant B.1.1.7 are located at sites of intermolecular interaction (4 out of 7 substitutions). One mutation (N501Y) enhanced the affinity of the spike protein with ACE2 [2]. N501Y was modeled based on the cryoEM structure of the Wuhan-Hu-1 spike protein/ACE2 complex [3] (Figure 2A, PDB 6M17), indicating that the gain in affinity likely results from aromatic interactions (π stacking) between Tyr501 and Tyr41 of ACE2 (Figure 2B).

Strikingly, 3 mutations in the spike protein that distinguish UK variant B.1.1.7 are located at interfaces between subunits of the trimeric protomer. Unlike the mutation at position 501 that increased affinity for ACE2, the 3 substitutions at spike trimer interfaces likely reduce intermolecular binding affinity. The mutations likely increase spike protein lability in a manner that enhances dynamic virus processes that include spike protein cleavage, structural rearrangement and host cell fusion mechanisms.

**Figure 3:**
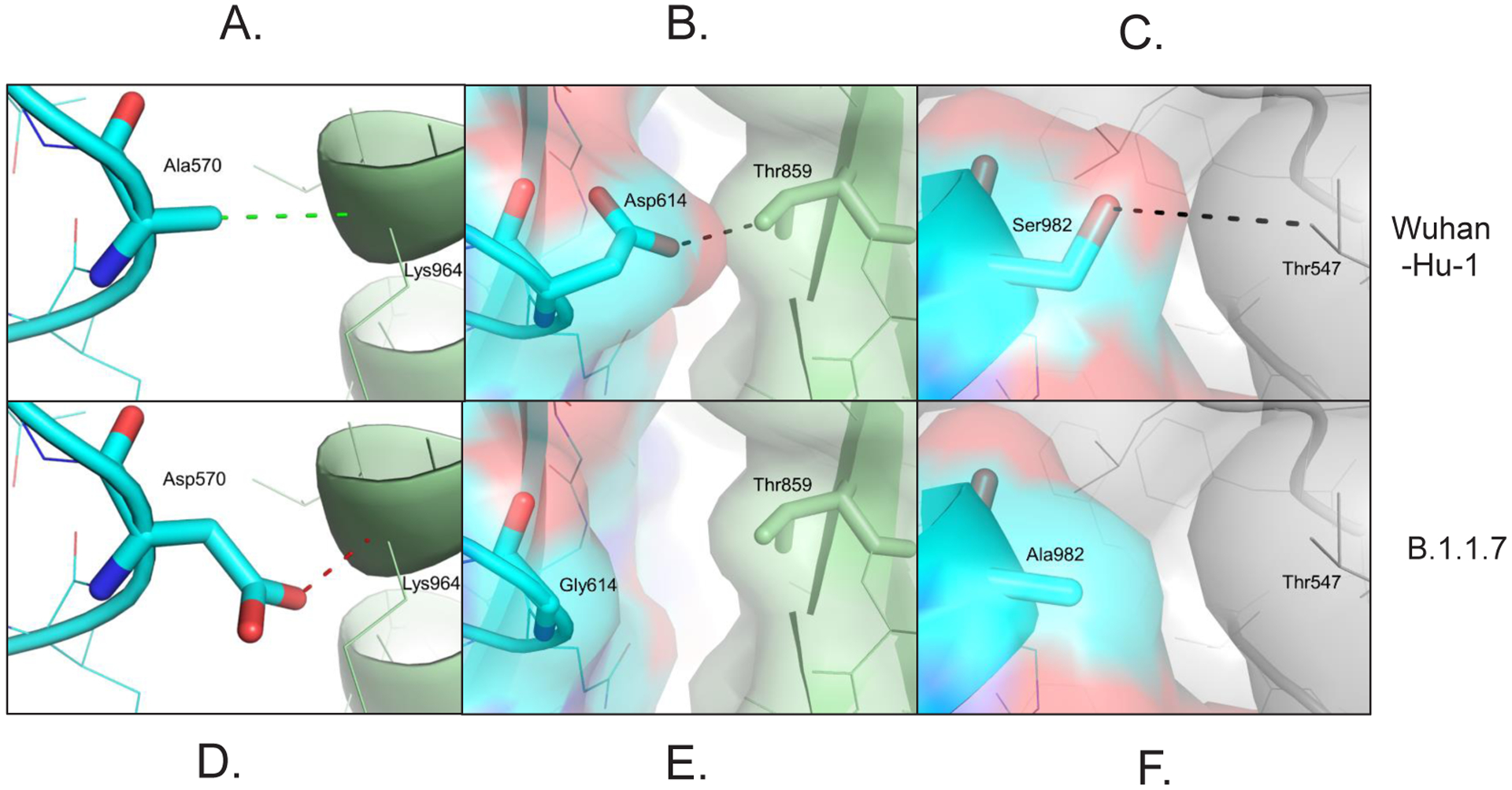
Mutations in the SARS-CoV-2 B.1.1.7 hinder intermolecular interactions between subunits of the spike glycoprotein. **A**. Ala570 in the spike glycoprotein from Wuhan-Hu-1 (blue) forms an intermolecular van der Waals (green dashes) contact with the main chain amide of Lys964 from the neighboring chain (grey). **B**. Asp570 in the SARS-CoV-2 UK variant is expected to form a repulsive interaction (red dashes) because of a potential clash with the main chain of the neighboring chain (grey). **C**. Asp614 in the spike glycoprotein from Wuhan-Hu-1 (blue) forms an intermolecular H bond with Thr859 a neighboring chain (green). **D**. Gly614 in the SARS-CoV-2 UK variant results in a loss of a H bond with Thr859 at the interface between two spike protein subunits (blue and green) of the trimeric protomer forming a potentially druggable structural pocket. **E**. Ser982 in the spike glycoprotein from Wuhan-Hu-1 (blue) forms an intermolecular H bond with Thr547 of a neighboring chain (grey). **F**. Ala982 in the spike protein of the UK variant (blue) prevents H bonding with Thr547 of a neighboring chain (grey).

Intermolecular interactions between individual chains of the SARS-CoV-2 spike glycoprotein were observed at positions A570 (Figure 3A), D614 (Figure 3B), and S982 (Figure 3C) in the original Wuhan strain (Wuhan-Hu-1). The A570D substitution in the UK variant B.1.1.7 variant introduces steric clash with the backbone amide of K964 (Figure 3D). The D614G substitution results in the formation of a distinctive cavity at the interface of spike protein subunits in the UK variant B.1.1.7 trimer (Figure 3E). The S982A in UK variant B.1.1.7 lacks intermolecular hydrogen (H) bonding potential between spike protein subunits at this site (Figure 3F). Collectively, these data suggest that: 1) the UK variant B.1.1.7 exhibits a change that enhances affinity for the coronavirus receptor ACE2 (N501Y), and 2) mutations may enhance dynamic virus fusion mechanisms by reducing intermolecular stability of spike protein subunits (A570D, D614G, S982A).

P681H represents a potentially important difference between Wuhan-Hu-1 sequence and UK variant B.1.1.7. Position 681 is located adjacent to the RRAR proprotein convertase motif considered a hallmark of high pathogenesis (**P**RRAR in Wuhan-Hu-1 [4], **H**RRAR in B.1.1.7). This site is cleaved by furin and other proteases to separate the S1 and S2 subunits of the spike protein, which undergo structural rearrangement and fusion with host cell membranes mediated by heptad repeat domains in S2. Since endosomal S1/S2 cleavage occurs in an acidified environment, a protonated histidine at position 681 of the UK variant B.1.1.7 has the potential to influence the rate of spike protein cleavage and subsequent membrane fusion mechanisms to gain cell entry.

**Figure 4:**
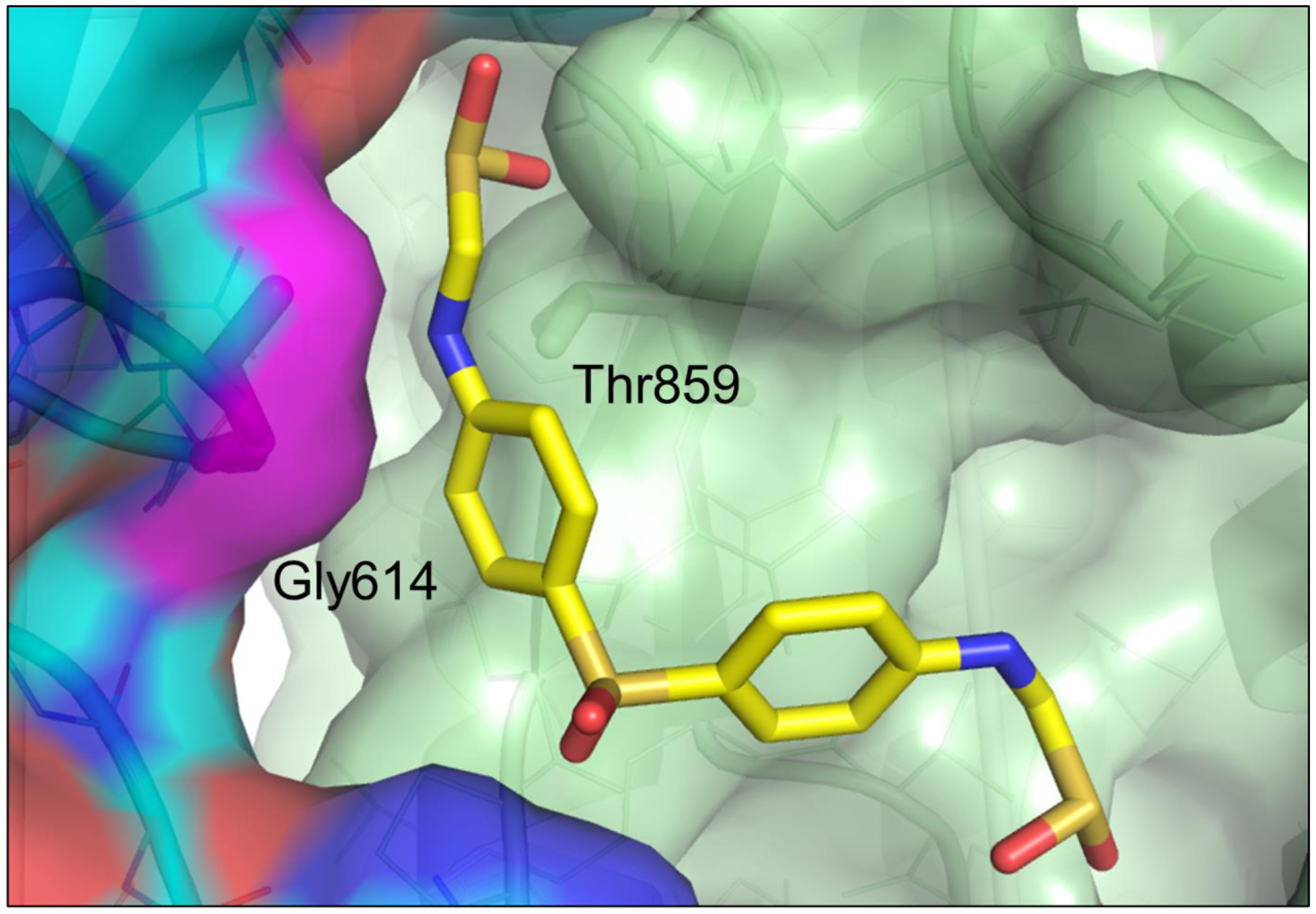
Highly transmissible strains of D614G SARS-CoV-2 in the global human population exhibit a unique, potentially druggable structural pocket. A model of the B.1.1.7 spike protein was used as the basis for molecular docking screening of 1,207 approved drugs. Sulfoxone was predicted by DOCK6.7 (UCSF) to bind the interface between subunits of the spike protomer at position 614 with an estimated ΔG value of −24.4 kcal/mol.

Since SARS-CoV-2 variants with D614G, such as UK variant B.1.1.7, currently predominate globally, we asked if the interface between individual chains of the spike protein trimer at position 614 may be druggable. A model of the spike protein trimer of the UK variant B.1.1.7 was used as the basis for molecular docking. 1,207 FDA approved drugs were docked to the interface site formed between position D614G and T859 of neighboring chains in the spike protein trimer. Drugs were estimated to bind the UK variant B.1.1.7 at the D614G site, such as the anti-leprosy drug sulfoxone, ΔG −24.4 kcal/mol (Figure 4). These data suggest that drugs may be developed to target highly transmissible SARS-CoV-2 strains including the UK variant B.1.1.7.

As mutations that distinguish the UK variant B.1.1.7 may influence immune responses, we analyzed solvent accessibility of positions that differ with Wuhan-Hu-1 (Table 1). Although N501Y has the potential to influence neutralizing antibody binding, this semiconservative difference is located at the edge of the ACE2/spike protein interface, therefore not expected to dramatically alter neutralizing antibody responses. T cells recognize SARS-CoV-2 peptides in the context of multiple HLA molecules [5], suggesting that differences between peptides derived from the Wuhan-Hu-1 spike protein or UK variant B.1.1.7 are not expected to radically influence the overall function of polyclonal T cell responsiveness to infection. However, surveillance of neutralizing antibody responses and T responses to peptides derived from the UK variant B.1.1.7 in COVID-19 patients will be required to understand functional effects on immune recognition.

Over the past year, the virus has adapted variant forms with improved fitness. Mutations that distinguish UK variant B.1.1.7 increase virus fitness through mechanisms that likely include: 1) increased affinity for binding to the coronavirus receptor ACE2, and 2) alteration of intermolecular contacts between subunits of the spike protein trimer. Changes in intermolecular contacts are expected to improve dynamic mechanisms involved in spike protein cleavage, structural rearrangement and host cell membrane fusion. Emerging mutations such as D614G can serve as the basis for drug discovery efforts to target specific highly transmissible variants such as UK variant B.1.1.7.

## Methods

### Sequences of UK variant B.1.1.7 and reference Wuhan-Hu-1

The Wuhan-Hu-1 sequence was used as a reference (GENBANK accession number MN908947). A Threat Assessment Brief, December 20, 2020, was used to define mutations that distinguish the UK variant B.1.1.7 (https://www.ecdc.europa.eu/en/publications-data/threat-assessment-brief-rapid-increase-sars-cov-2-variant-united-kingdom).

### Modeling mutations in the UK variant B.1.1.7 spike glycoprotein

The cryoEM structure of the SARS-CoV-2 (Wuhan-Hu-1) RBD/ACE2-B0AT1 complex [3] (PDB 6M17) was used as the basis for modeling N501Y. The prefusion SARS-CoV-2 (Wuhan-Hu-1) spike glycoprotein with a single receptor-binding domain up [6] (PDB 6VSB) was used as the basis for modeling D570A, D614G, T716I, S982A and D1118H. Side chains were mutated in COOT [7] using rotamers that represent a local energy minimum of torsional angles.

### Molecular docking

We used the atomic model of the UK variant B.1.1.7 spike protein as the basis for molecular docking. To prepare the site for docking, all water molecules were removed. We explored the molecular surface of the structure using sets of spheres to describe potential binding pockets at the UK variant B.1.1.7 interface between the equivalent of chains A and B of PDB 6VSB. The sites selected for molecular docking were defined using the SPHGEN program in DOCK [8,9], which generates a grid of points that reflect the shape of the selected site, then filtered through CLUSTER. The CLUSTER program groups the selected spheres to define the points that were used by DOCK6.7 (UCSF) to match potential ligand atoms with spheres. Intermolecular AMBER energy scoring (van der Waals plus columbic), contact scoring, and bump filtering were implemented in the DOCK program algorithm. Atomic coordinates for 1,207 FDA approved small were positioned in a structural pocket located at position G614 of chain A, and position T859 in chain B. Each drug was docked in 1,000 different orientations and scored on the basis of predicted polar (hydrogen bond) and nonpolar (van der Waals) interactions. The most favorable orientation and scores (contact and electrostatic) were calculated. PyMOL (https://pymol.org/2/) was used to generate molecular graphic images and animation (Supplemental material).

## Supplementary Material

JCI-20-085_Supplementary_file

## Figures and Tables

**Table 1:** Locations, surface accessibility and interactions of mutations that distinguish the B.1.1.7 and Wuhan-Hu-1 spike proteins.

Mutation	Location	Interaction	Solvent accessibility in protomer (PDB 6VSB)	Potential effect
Deletion 69–70	N-terminal domain	None	None	Loss of T and B cell epitopes
Deletion 144–145	N-terminal domain	None	None	Loss of T and B cell epitopes
Asn501Tyr	RBD	Lys 353 in ACE2 PDB 6M17	Exposed	Enhance ACE2 binding affinity Altered T and B cell epitopes
Ala570Asp	Between RBD and S1/S2 boundary	Intermolecular contact with Lys 964, (chain B 6VSB)	Exposed	Protomer stability, dynamics of cleavage and fusion Altered T and B cell epitopes
Asp614Gly	Between RBD and S1/S2 boundary	Intermolecular contact with Thr 859 (chain B 6VSB)	Exposed	Protomer stability, dynamics of cleavage and fusion Altered T and B cell epitopes
Pro681His	Adjacent to furin cleavage site	None	Exposed	Altered S1/S2 cleavage in endosomes from protonated His Alteration of T and B cell epitopes
Thr716Ile	S2 between fusion peptide and Heptad Repeat 1	None	Exposed	Minor alteration of T and B cell epitopes
Ser982Ala	Heptad Repeat 1 in S2	Intermolecular contact with Thr 547 (chain C 6VSB)	Exposed	Protomer stability, dynamics of cleavage and fusion Alteration of T and B cell epitopes
Asp1118His	S2	Intramolecular contact with Val 951	Buried	Alteration of T cell epitopes
